# Refined on-tissue chemical derivatization expands MALDI-MSI capabilities for spatial metabolomics of mammalian organs and metastatic tumors using EDC and 4-APEBA

**DOI:** 10.1186/s12935-026-04494-x

**Published:** 2026-11-04

**Authors:** Antonia Fecke, Luiza Martins Nascentes Melo, Ann-Cathrin Groba, Gabriele Allies, Feyza Cansiz, Siva Swapna Kasarla, Sven Heiles, Albert Sickmann, Alpaslan Tasdogan, Karl W. Smith

**Affiliations:** 1 Leibniz-Institut für Analytische Wissenschaften—ISAS—e.V, Otto-Hahn-Str. 6b, 44227 Dortmund, Germany; 2 Department for Dermatology, Venerology and Allergology, Research Center One Health, University Hospital Essen, German Cancer Consortium, Partner Site Essen & University Alliance Ruhr, NCT West, Campus Essen, 45147 Essen, Germany; 3 Lipidomics, Faculty of Chemistry, University of Duisburg-Essen, 45141 Essen, Germany; 4 Medizinische Fakultät, Medizinische Proteom-Center (MPC), Ruhr-Universität Bochum, 44801 Bochum, Germany

**Keywords:** On-tissue chemical derivatization, MALDI-MSI, Spatial metabolomics, Metastasis

## Abstract

**Background:**

Matrix-assisted laser desorption/ionization mass spectrometry imaging (MALDI-MSI) is widely used in metabolomics because it enables high-resolution spatial characterization of molecular distributions directly from tissue. Nevertheless, comprehensive analysis of small, polar metabolites remains difficult due to limited ionization efficiencies and diverse chemical structures. On-tissue chemical derivatization (OTCD) has emerged as an effective strategy to improve ionization efficiency, sensitivity, and selectivity for these analytes.

**Methods:**

In this study, we established and systematically optimized an OTCD approach for spatial analysis of carbonyl- and carboxyl-containing metabolites in murine tissues using 4-APEBA in combination with carbodiimide crosslinker EDC. Reagent concentrations, solvent composition, and spray parameters were optimized to achieve reproducible derivatization across multiple tissue types.

**Results:**

The optimized protocol enabled reproducible and confident detection of carbonyl- and carboxyl-containing metabolites in positive ion mode through the characteristic bromine isotopic signature of 4-APEBA. Application to metastatic liver and lung tissue demonstrated the method’s performance in biologically complex environments. Pronounced metabolic heterogeneity correlated with histopathological features in tumor-rich liver sections, and clear tumor-specific localizations were obtained in lung tissue with only small metastatic foci. Metabolic differences between tumor and adjacent healthy regions were consistent with established cancer metabolism pathways, including glycolytic reprogramming.

**Conclusion:**

This study is the first to optimize and investigate sample preparation conditions for mouse tissue application of a reliable OTCD workflow using EDC and 4-APEBA for MALDI-MSI-based spatial metabolomics, with localized OTCD-MSI of melanoma metastases. By utilizing this combination of reagents, carbonyl and carboxyl moiety containing molecules are confidently annotated based on isotopic signatures in positive mode ionization. The application is capable of interrogating metabolic heterogeneity in clinically relevant tumor models and demonstrates its value for future investigations of tumor metabolism.

**Graphical Abstract:**

**Figure:**
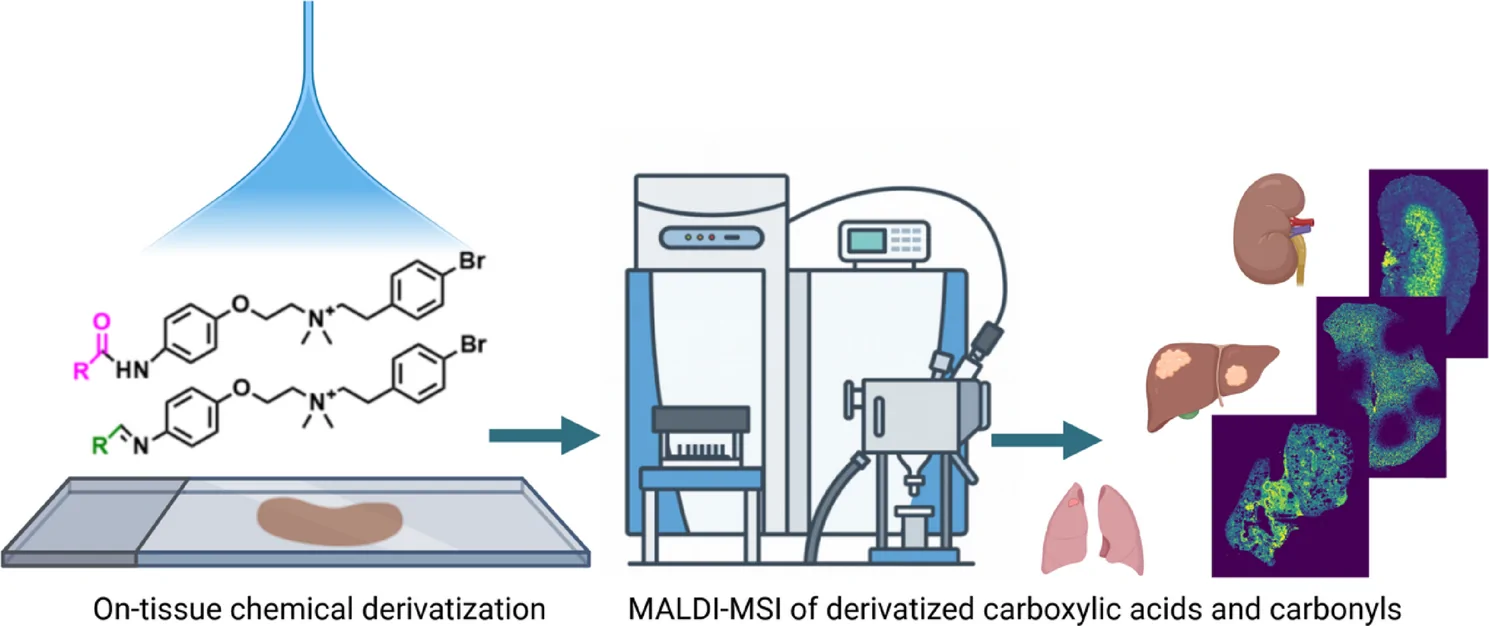

**Supplementary Information:**

The online version contains supplementary material available at https://doi.org/10.1186/s12935-026-04494-x.

## Background

Spatial small-molecule metabolite (SMM) analysis has become increasingly important due to its ability to provide functional insights into cellular processes, biological pathways, and disease mechanisms [1]. Metabolites are key intermediates and end products of metabolism, contributing to signaling, energy production, and physiological regulation [2]. Spatially resolved analytical technologies enable the investigation of localized metabolic processes and tissue heterogeneity that are not accessible through bulk extraction-based methods such as liquid chromatography–tandem mass spectrometry (LC-MS/MS). These approaches provide unique opportunities to examine metabolic alterations in pathological regions, such as tumor microenvironments or sites of cardiovascular disease [3–5].

Matrix-assisted laser desorption/ionization mass spectrometry imaging (MALDI-MSI) is one of the most widely used platforms for spatial molecular analysis. It enables label-free detection of diverse biomolecular classes, including glycans [6], lipids [7, 8], proteins [9], and metabolites [10, 11]. Although sensitivity and coverage for small metabolites have improved through instrumental and methodological advances [11–14], significant analytical challenges remain, including metabolite identification. Metabolite identification is a common challenge as a whole in metabolomics, including LC-MS/MS [15]. The broad physicochemical diversity of metabolites and their variable ionization efficiencies lead to incomplete coverage [16]. Low abundance, ion suppression in the low *m/z* range, and reduced ion yield in high-resolution MSI further complicate the detection of many metabolites [17]. As a result, only a fraction of the metabolome is routinely visualized in standard MALDI-MSI experiments.

On-tissue chemical derivatization (OTCD) has emerged as a promising strategy to address these limitations by introducing readily ionizable moieties or permanent charges into target analytes, thereby increasing ionization efficiency, sensitivity, and analytical selectivity [18]. Additionally, chemical tagging increases the molecular mass of small molecules, helping reduce spectral interference from matrix-related ions [19].

Multiple derivatization reagents with different target classes and reaction mechanisms have been employed for OTCD-MALDI-MSI. 2-fluoro-1-methylpyridinium (FMP-10) represents a reactive matrix used for derivatization of primary and secondary amines and phenol compounds and is used to map neurotransmitters and small related molecules [20, 21]. Girard’s T and P reagents are well-established, hydrazine-based reagents that target carbonyl-containing compounds such as steroids and neutral sterols [22–26]. Carboxylic acids likewise have been targeted by several OTCD reagents, including 2-picolylamine, which has been demonstrated for fatty acids [27]. More recently, *N*,*N*,*N*-trimethyl-2-(piperazin-1-yl)ethan-1-aminium iodide (TMPA) was developed and used to derivatize fatty acids, bile acids and TCA-cycle acids in kidney tissue, demonstrating the applicability of OTCD to small acidic compounds [28]. While these approaches for carbonyl- and carboxylic acid derivatization target individual functional groups, derivatization strategies with broader class coverage have been established. 1-(4-(aminomethyl)phenyl)pyridin-1-ium chloride (AMPP) for example enables charge-tagging of both carboxylic acids and carbonyl-containing metabolites [29].

Recently, a two-step OTCD approach combining the carbodiimide crosslinker 1-ethyl-3-(3-(dimentyhlamino)propyl)carbodiimide (EDC) with 4-APEBA — 4-(2-((4-bromophenethyl)dimethylammonio)ethoxy)benzenaminium dibromide — has demonstrated broad utility for derivatizing both carbonyl- and carboxyl-containing metabolites in plant tissue, agar, and human placenta [30–32]. This workflow enables detection of central metabolic intermediates, such as tricarboxylic acid (TCA) cycle metabolites, which play pivotal roles in energy production and cellular signaling [33]. A further key advantage of 4-APEBA derivatization is the incorporation of a bromine-containing moiety that produces distinctive isotopic doublets due to the ~ 1:1 abundance of ^79^Br and ^81^Br, thereby increasing annotation confidence in untargeted metabolomics [34]. To date, this workflow and the associated reagents has only been optimized on plant material, evaluated on limited sample types and have been successfully implemented in a single laboratory using specific instrumentation. Despite the availability of detailed methodology and well-designed procedures, the method reported from literature was not readily transferable to instrumentation not explicitly used in the described workflow [35]. Furthermore, although the EDC/4-APEBA derivatization strategy offers analytical advantages, it has not previously been optimized for across a broader range of MALDI-MSI instrumentation, nor has it been applied to widely used mammalian animal disease models, such as murine cancer tissues.

In this study, we systematically optimized an OTCD workflow for enhanced spatial analysis of carbonyl- and carboxyl-containing metabolites in heterogeneous mouse tissue from healthy and cancer affected organs. We evaluated the effects of reagent concentration and solvent composition to maximize metabolite coverage and ion yields compared with conventional sample preparation. The optimized OTCD-MALDI-MSI method was applied to healthy kidney tissue, and to mouse liver and lung tissues containing metastatic melanoma lesions. These applications demonstrate reproducible performance of signal intensity, metabolic coverage and spatial orientation of analytes in both tumor-rich samples and highly heterogeneous tissues, enabling characterization of metabolic differences between tumor and surrounding healthy regions. Overall, this work expands the applicability of OTCD-MALDI-MSI-based metabolomics by providing a reproducible workflow for the detection of carbonyl- and carboxyl-containing metabolites and fatty acids in positive ion mode with characteristic bromine isotopic signals for increased annotation confidence. In addition, the characteristic mass shift introduced by derivatization further improves annotation confidence by separating derivatized metabolites from endogenous and matrix-introduced background interference. Thereby, it supports the investigation of metabolite-level heterogeneity in biologically and clinically relevant tissue environments.

## Materials and methods

### Materials

The MALDI matrix 2,5-dihydrobenzoic acid (DHB) and 1-ethyl-3-(3-(dimentyhlamino)propyl)carbodiimide (EDC) were purchased from Sigma-Aldrich (Darmstadt, Germany). 4-(2-((4-bromophenethyl)-dimethylammonium)ethoxy) benzenaminium dibromide (4-APEBA) was purchased from Axon Medchem BV (Groningen, The Netherlands). LC-MS grade solvents acetonitrile (ACN) and methanol (MeOH) were purchased from Biosolve BV (Valkeswaard, The Netherlands). Ethanol (EtOH), Mayer’s hematoxylin, Eosin Y (0.5% alcoholic solution), xylene, and Histomount were all purchased from Sigma-Aldrich (Darmstadt, Germany). LC-MS grade water was obtained from Carl Roth (Karlsruhe, Germany). Metabolite standards were bought from MetaSci (Toronto, Canada). Deuterated creatine (d3) was obtained from Cambridge Isotope Laboratories Inc. (Andover, MA, USA).

### Mouse model

In this study, wild-type B6N.129S6 male mice were used. Mice breeding, housing, and organ collection were performed in accordance with the guidelines from Directive 2010/63/EU of the European Parliament on the protection of animals used for scientific purposes. Organ removal was performed for scientific purposes according to § 4 of the Animal Welfare Act (Tierschutzgesetz; TierSchG), with all animals recorded, documented, and reported under the German Decree on the Reporting of Laboratory Animals (Versuchstier-Meldeverordnung, VersTierMeldV). Mice were sacrificed by cervical dislocation at an age of 7–8 months. Organs were extracted, snap-frozen in liquid nitrogen, and stored at − 80 °C until MSI analysis.

All mice in the Central Animal Laboratory of Essen University Hospital (Zentrales Tierlaboratorium, ZTL) are kept under specified pathogen-free (SPF) conditions in individually ventilated cage systems (IVCs). All mouse housing facilities are climate-controlled in accordance with the requirements of Annex III of Directive 2010/63/EU, ensuring a constant temperature of 22 °C ± 2 °C and a defined relative humidity of 55 ± 10%. The light/dark cycle is 12:12. Food and water are offered ad libitum. Health monitoring is carried out in strict accordance with FELASA recommendations four times a year.

The mouse experiment with A375 human melanoma cells and human melanoma patient-derived xenograft (PDX) M481 was conducted in accordance with relevant ethical regulations and approved by the local ethics committee in compliance with the European Convention for the Protection of Vertebrate Animals Used for Experimental and other Scientific Purposes (Directive 2010/63/EU, 81-02.04.2023.A026 Landesamt für Natur, Umwelt und Verbraucherschutz Nordrhein-Westfalen, Germany). NOD-*Prkdc*^*scid*^*-IL2rg*^*Tm1*^/Rj (NXG) (RRID: IMSR_RJ: NXG) mice were purchased from Janvier Labs (France). Throughout the study, mice were maintained on standard chow and provided food and water ad libitum. A375 human melanoma cells and human melanoma PDX M481 cells were injected into 5-8-weeks-old female NXG mice. Briefly, melanoma cells suspension was prepared in RPMI medium supplemented with 10% FBS, 1% penicillin/streptomycin with Matrigel (1:1 ratio matrigel/cells) (Corning, CLS356234). Subcutaneous injections were performed in the right flank of mice in a final volume of 100 µL using 1 × 10^4^ cells. The maximum allowable tumor diameter of 2.0 cm was strictly observed and not exceeded in any experiment. At that maximal ethical endpoint, all mice in the cohort were euthanized, per approved protocol, for analyses of metastatic disease burden from distant organs.

### Tissue sectioning

For cryosectioning of the organs prior to MALDI-MSI analysis, the tissue was placed into a Leica CM1860 Cryostat chamber for 30 min of equilibration to the chamber temperature. Deionized water was used to mount the tissue to cryosectioning chucks. Tissue sections of 12 μm thickness were taken at -20 °C. All sections used for MALDI-MSI were thaw mounted to ITO slides (70–100 ohms, Delta Technologies, USA). For lung tissue, serial sections were collected on regular glass slides for histological staining. Sections were vacuum packed and stored at -80 °C until further used. For each organ (kidney, liver containing metastasis and lung containing metastasis), one specimen was used, and serial sections were used as technical replicates.

### In-solution derivatization experiments

Separate stock solutions of pyruvate, vanillin, citric acid, fumaric acid, glucose and alpha-ketoglutaric acid were dissolved in water (0.01 mg/mL and 1 mg/mL in water) and were aliquoted (5 µL) into an Eppendorf vial. 20 µL of EDC (6 mg/mL in water) and 4-APEBA (2 mg/mL in water) were added respectively. The samples were vortexed for 2 h at 4 °C, 40 °C, and room temperature in different experiments. After the reaction time was complete, the derivatized standards were mixed 1:1 with DHB (40 mg/mL 70:30; MeOH: H_2_O) and 1 µL of this mixture was spotted onto tinfoil-covered ITO slides. The spots were dried before subsequent MALDI analysis.

### On-tissue chemical derivatization and matrix application

For on-tissue chemical derivatization, sectioned samples were dried for 40 min in a vacuum desiccator to remove excess moisture to avoid analyte delocalization. Sections were then sprayed with the derivatization agents (DAs) using a SunCollect Sunchrom sprayer. Optimization for mammalian tissue samples was performed using several concentrations of 4-APEBA (1 mg/mL, 0.5 mg/mL), layers of 6 mg/mL EDC (4,8 and 12), and solvent combinations (100% H_2_O,50:50 ACN: H_2_O). The reagents with their applicable parameters have been evaluated to replicate tissue sections and assessed based on spatial localization, signal intensity, and metabolic coverage. The optimal parameters were chosen as follows and applied to all following experiments: 8 layers of 6 mg/mL (50:50 ACN: H_2_O) EDC followed by 4 layers 0.5 mg/mL (50:50 ACN: H_2_O) 4-APEBA sprayed at 20 µL/min and a nozzle position of z = 20 (arbitrary value). The MALDI matrix DHB was sprayed after the application of DAs in 15 layers (15–40 µL/min) at 40 mg/mL (70:30 MeOH: H_2_O) for kidney and lung tissue and 26 layers (20 µL/min) at 20 mg/mL (90:10 MeOH: H_2_O + 0.1% trifluoroacetic acid (TFA)) for liver tissue. All reagents are freshly prepared prior to the experiment and tissue sections are measured on the same day of sample preparation, in order to reduce any instability or further reactive inconsistencies.

To compare metabolic coverage to traditional matrices, kidney sections were prepared. For each section, one half of the tissue was derivatized using the optimized OTCD-protocol and the other half was covered with a cover slip. After completion of the OTCD procedure, the reverse half of the tissue was sprayed with either DHB (15 layers, 40 mg/mL, 70:30 MeOH: H_2_O, see above) or N-(1-naphthyl)ethylenediamine dihydrochloride (NEDC, 7 mg/mL, 70:25:5 MeOH: ACN: H_2_O, layer 1–8: 15µL/min, layer 9–20: 20 µL/min).

### Derivatization of dried droplet spots

For confirmation of annotated metabolites, selected analytical standards of lactic acid, pyruvate, alpha-ketoglutaric acid, glutathione, citric acid, aconitic acid, creatine, malic acid, succinic acid and fumaric acid were spotted onto an ITO-slide (1 µL at a concentration of 0.1, 0.05 and 0.025 mg/mL) and dried under vacuum. Slides were then sprayed using the described optimized protocol and were analyzed to investigate successful derivatization products and fragmentation patterns in MS/MS. Additionally to investigate short-term stability of derivatization products, 3 µL of analytical standards lactic acid, citric acid, malic acid, aconitic acid, glutathione reduced, pyroglutamic acid, hydroxybutyric acid, ketobutyric acid, fumaric acid, succinic acid and pantothenic acid (0.1 mg/mL) were spotted onto ITO slides in triplicate. The slides were then sprayed with the optimized OTCD-protocol, and scans were collected from three areas in the spot on three consecutive days to compare signal-to-noise ratios over time.

### Derivatization yield experiment

To investigate the reaction yield of the on-slide spray reaction, deuterated creatine (d3-creatine) has been utilized as a metabolite standard detectable with and without derivatization. The standard was prepared in a concentration of 10 µg/mL in 50:50 ACN: H_2_O and sprayed onto an ITO-slide (20 layers, 20 µL/min). Half of the coated slide was covered with EDC and 4-APEBA based on the aforementioned protocol, the other half of the slide was sprayed only with DHB. Defined areas have been measured and analyzed based on the detection of derivatized or underivatized d3-creatine.

### MALDI-MSI experiments

All MSI experiments were performed on an Orbitrap Q-Exactive HF mass spectrometer (Thermo Fisher Scientific GmbH, Bremen, Germany) coupled to an elevated pressure MALDI ion source (Spectroglyph LLC, Kennewick, WA, USA) [36]. The 349 nm MALDI Nd: YLF laser (Explorer One, Spectra Physics, Mountain View, CA) was operated at a repetition rate of 500 Hz and pulse energy of 1–2 µJ. The laser was focused to a spot size of 15 μm, and the pixel sizes for analyses were 50 × 50 μm for the kidney section, 20 × 20 μm for the lung samples and 25 × 25 μm for the metastatic liver samples. Liver, lung and final kidney sections were measured in serially sectioned triplicate. The system was operated at ∼6.5 Torr. In positive ion mode, the high-pressure funnel (HPF) and low-pressure funnel (LPF) were operated at 225 V_pp_ and 125 V_pp_, respectively, achieved by using 35% RF drive and 30% RF drive for 720 kHz and 825 kHz, respectively. In negative mode, the HPF and LPF were operated at 98 V_pp_ and 75 V_pp_, respectively, achieved by using 15% RF drive for 720 kHz and 825 kHz, respectively. The mass spectrometer was operated in the mass range *m/z* 300–1000 for general OTCD measurements, *m/z* 100–1000 for d3-creatine experiments, standard stability measurements and matrix comparison experiments and *m/z* 50–750 for NEDC and DHB experiments with the automatic gain control (AGC) mode turned off. Resolution was set at 120,000 for *m/z* 200 and a set inject time of 250 ms was used, resulting in 125 shots/pixel and ∼3 scans/second. Mass calibration in positive ion mode was performed with an electrospray ionization source using Pierce positive Ion CalMix calibration solution (Thermo Fisher Scientific), according to the manufacturer’s instructions. In negative mode, instrument calibration was performed using a Pierce Negative Ion Calmix solution spiked with metabolite standards (caffeic acid, lactic acid, pyruvate, taurine, uridine-phosphoric acid, glutathione, and citric acid) to cover the low mass range targeted in this method. For tandem mass spectrometry (MS/MS) analysis, an isolation window of 0.5 Da was used with varying collision energies (Supplementary Table 1).

### MALDI-MSI data processing

Thermo RAW (.raw) and positional files (.xml) were uploaded to the built-in converter for LipostarMSI, which utilizes msconvert from ProteoWizard (3.0.22317) for initial conversion to imzML format [37]. The imzML file was then loaded into LipostarMSI using the following parameters: 5 ppm *m*/*z* tolerance, peak intensity > 1%, peak detection frequency > 1% and minimum spatial chaos > 0.7. Hotspot removal was applied using the 99% quantile and no denoising was applied for all tissue samples. Hotspot removal was not applied for dried droplet experiments. All images were normalized to the total ion current (TIC). Comparisons of signal intensities were performed by manually drawing regions of interest (ROIs) of the complete tissue (kidney) or tumor and healthy tissue areas (liver metastases) in LipostarMSI and extracting average signal intensities from these ROIs. Statistical comparisons between tumor and healthy ROIs were done using an ordinary one-way-ANOVA followed by Dunnett’s multiple comparison test. A p-value of 0.05 was considered statistically significant. Statistical analysis was performed using GraphPad Prism (v. 10.0.3 (275)). Stability data of standard experiments and MS/MS data were processed using Thermo Xcalibur Qual Browser (v. 4.4.16.14). To calculate background interference, average signal intensities were extracted from on-tissue and off-tissue ROIs at the respective *m/z* values. Relative off-tissue background was calculated as *Intensity off-tissue/Intensity on-tissue × 100*. Additionally, single-pixel S/N (signal-to-noise) was extracted in Qual Browser.

Annotations were performed via manual curation of the data and a simple Python (version 3.9) pipeline. First, *m/z* values of all picked peaks were extracted and isotope peak pairs with 1.998 Da difference were identified. All peak pairs were manually curated with a focus on signal localization (on or off tissue) and signal intensity. Proposed ^79^Br containing masses of derivatives were extracted and tentatively matched to metabolites of the Human Metabolome Database (HMDB) based on theoretical mass (± 5 ppm window), functional groups and biological origins of analytes, corresponding to a level 2–3 annotation confidence [38]. Pathway classification was performed using the Pathway Analysis tool in MetaboAnalyst 6.0. Classification into chemical classes was performed using ClassyFire Batch by Fiehn Lab. All annotations are tentative based, with isobaric and isomeric compounds not fully possible to distinguish in this method, as common with MALDI-MSI analysis.

For segmentation analysis, the measured MSI data were imported from the paired .imzML and .ibd files using the pyimzML package. All spectra were binned along the *m/z*. Spectral intensities were normalized by the TIC, and signals below a low-intensity threshold were set to zero to suppress residual noise. Log-transformation was applied to stabilize intensity variance, and spatial smoothing using k-nearest neighbor averaging (k = 8) was applied to reduce technical noise prior to multivariate analysis.

For dimensionality reduction and visualization, principal component analysis (PCA) followed by uniform manifold approximation and projection (UMAP) was performed. Unsupervised clustering algorithms (k-means, hierarchical, and spectral clustering) were applied to the PCA-reduced data, and the resulting segmentations were projected onto both the UMAP embedding and the original spatial image grid. Internal clustering validation metrics were computed to evaluate segmentation quality and to help with the selection of the optimal number of clusters. Optimal number of clusters was calculated using silhouette Score, Davies-Bouldin index and Calinski-Harabasz index. For this analysis Python (3.13.13) and the scikit-learn (1.9.0) package were used. Quantitative agreement between annotated H&E images and spatial segmentation was evaluated within tissue-regions common to both modalities by using confusion matrices, overlay maps and DICE-coefficients. Image registration and binary mask generation were performed using Fiji (ImageJ 1.54p) and quantitative overlap analysis was performed in Python. The full list of Python packages used can be found in Supplementary Table 2.

### H&E staining

After MALDI-MSI analysis, analyzed sections were stained with hematoxylin and eosin (H&E). Samples were first immersed in 95% Ethanol for 30s to remove derivatization agents (DAs) and the MALDI matrix. Next the tissue was fixated for 3 min in phosphate buffered saline (PBS), immersed in diluted hematoxylin (1:2 dilution in 100% ethanol) for 1 min, bluing under tap water for 1 min, and 0.25% eosin Y solution for 2 min, followed by a series of 15 s of dH_2_O, 15 s 70% EtOH, 15 s 80% EtOH, 15 s 90% EtOH, 3 min for 100% EtOH and xylene for 3 min. Histomount was then used to fix the slides with a coverslip. Microscopy images were captured using brightfield Keyence VHX-7000 N (Keyence Deutschland GmbH, Frankfurt am Main, Germany). Serial stitched images were performed using the 200× magnification.

## Results

### In-solution reaction of OTCD reagents and metabolite standards

Preliminary experiments, as common to OTCD workflow developments, were performed in solution to assess the general reactivity of EDC and 4-APEBA with carboxylic acids and carbonyls with our instrumentation. Six metabolite standards (pyruvate, vanillin, citric acid, fumaric acid, glucose and α-ketoglutaric acid) were selected based on their functional groups and pre-existing data from literature. They were utilized for reaction with 4-APEBA and EDC and then spotted onto a tinfoil-covered glass slide. The anticipated derivatization products based on theoretical reactions with the expected two mass shifts were only consistently achieved for pyruvate and vanillin. Additional testing with higher standard concentrations (1 mg/mL) and varying temperatures (4 °C, 20 °C, 40 °C) showed no significant improvement in sensitivity and derivatization of carboxylic acids remained limited (Supplementary Figure S1). Both pyruvate and vanillin are carbonyl-containing metabolites, suggesting that the activation step using EDC needed for the successful derivatization of carboxylic acids may be the limiting factor [39]. However, as the influence of pH was not systematically investigated in this study, an impaired EDC-mediated activation step remains a hypothetical mechanism.

### Optimization for the application of EDC and 4-APEBA on-tissue

**Fig. 1:**
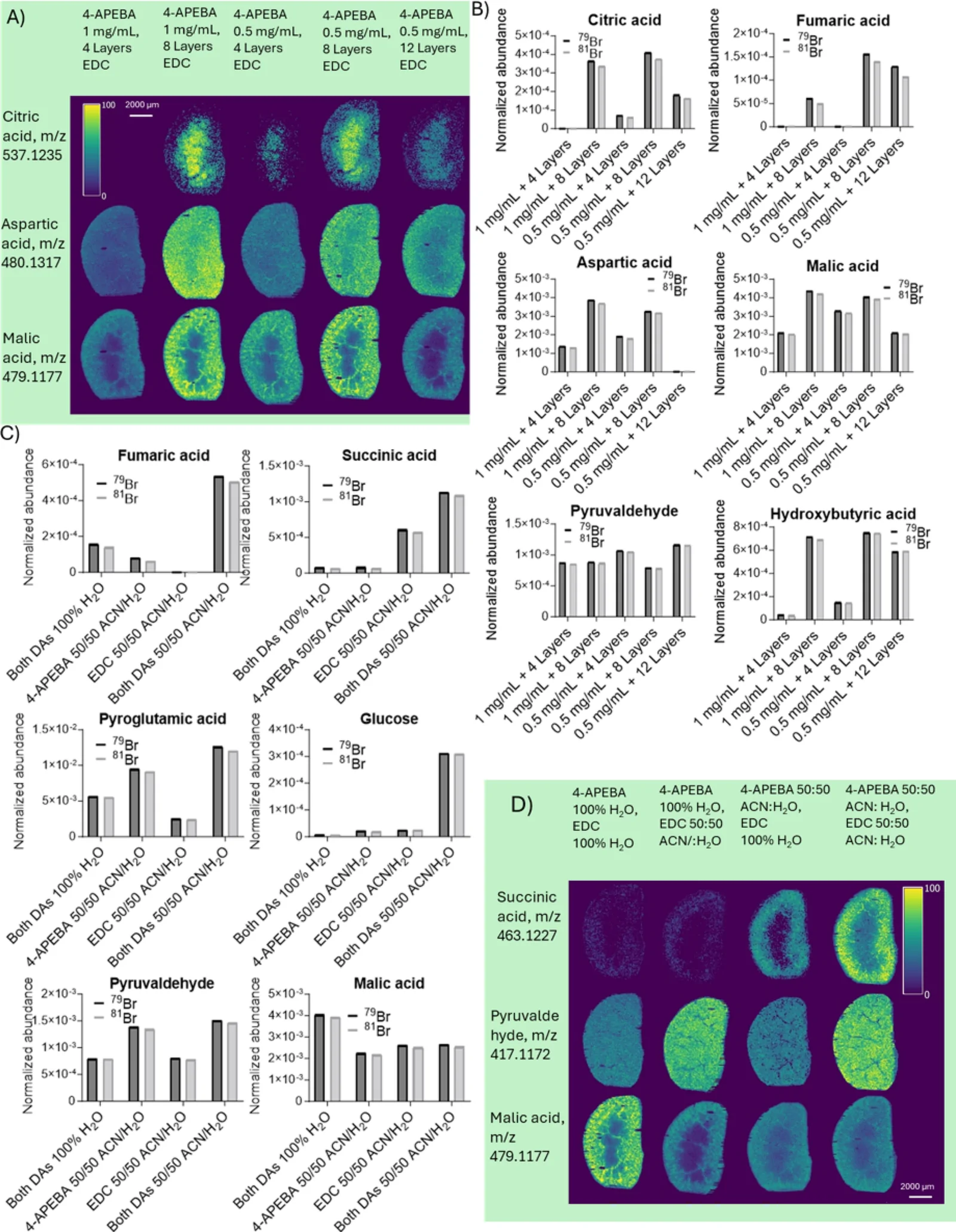
Optimization of the on-tissue derivatization reaction using EDC and 4-APEBA in mouse kidney tissue. **A**) Comparative MALDI ion images of selected metabolites under five different derivatization conditions. The concentration of 4-APEBA and the number of EDC spray layers were systematically varied. Signal intensities are indicated by the color scale. **B**) TIC-normalized signal intensities of selected metabolites derivatized using different 4-APEBA concentrations and numbers of EDC layers, displayed as bar plots. **C**) TIC-normalized signal intensities of selected metabolites derivatized using different solvent compositions for EDC and 4-APEBA, displayed as bar plots. **D**) Comparative MALDI ion images of selected metabolites under four different solvent compositions for EDC and 4-APEBA. Signal intensities are indicated by the color scale. Metabolite annotations were performed using a ± 5 ppm mass error tolerance

To establish a derivatization protocol on mouse tissue, we first attempted reproducing existing protocols for DA application and adapted them to our instrumentation [35]. Using reported reaction conditions, we successfully derivatized metabolites on kidney sections; however, substantial differences were observed in terms of signal intensity, metabolic coverage, and image quality compared to the literature, likely due to usage of different spraying device and sample type [30–32]. The previously published protocol was therefore not directly transferable to our instrumentation and required systematic re-optimization of application and derivatization conditions. A comparison summary between previously established parameters and parameters used in this study, including the rationale for any modifications, can be found in Supplementary Table 3. To improve the reaction for mouse tissue using the SunChrom sprayer, we first varied the concentration of 4-APEBA (1 mg/mL, 0.5 mg/mL) and the number of layers of 6 mg/mL EDC (4, 8 and 12 layers). Decreasing the concentration of 4-APEBA to 0.5 mg/mL resulted in a lower base peak of the derivative in the mass spectrometer and so aided in the analysis of relative intensities. This did not decrease the number of annotations or sensitivity of metabolites significantly (Fig. 1A and B). Therefore, higher concentrations were deemed unnecessary, and 0.5 mg/mL was chosen as the optimal concentration for OTCD, ensuring high sensitivity and coverage while avoiding oversaturation of the mass analyzer or signal suppression. We next optimized the number of layers of EDC applied to the tissue as we observed low signal intensities and limited coverage of derivatized carboxylic acids, compared to previously published literature An improvement in both parameters using 8 instead of 4 layers of 6 mg/mL EDC was observed, however a further increase in the amount of EDC applied did not lead to further signal improvement and was therefore deemed unnecessary. The optimization experiment tests revealed that parameter variation influences individual analytes differently, with some metabolites showing higher signal intensity under alternative conditions. For example, malic acid and aspartic acid showed slightly higher signal intensities across the tissue when using 1 mg/mL 4-APEBA and 8 layers of EDC. Other metabolites, such as pyruvaldehyde, do not seem to be affected significantly by these changes. The chosen parameters of 0.5 mg/mL 4-APEBA and increased spray layers of EDC (8 layers at 6 mg/mL) yield a balanced performance for robust detection of the intended polar metabolite targets on mouse tissue (Fig. 1A and B).

Additionally, we tested the influence of DA solvent compositions on coverage, sensitivity and, importantly, on spray and image quality. The original protocol from Zemaitis et al. [35] suggests spraying both DAs in pure aqueous solutions and notes that the inclusion of methanol resulted in fewer metabolite annotations in their tested samples. However, spraying the reagents in only water leads to very wet spraying and, therefore, occasional delocalization and inhomogeneous tissue coverage when using a sprayer with no heated nozzle. Among the tested conditions, using 50:50 ACN: H_2_O for both DAs showed the most favorable results, especially in terms of spray and image quality. Using 50% ACN resulted in fine and homogeneous spray results and hindered delocalization of water-soluble metabolites, as there was no obvious penetration of outer tissue boundaries for the highlighted molecules. However, the solvent composition can influence the relative performance of individual metabolites, with some analytes showing enhanced signal in aqueous conditions while others benefit from the organic content. Metabolites like fumaric acid, succinic acid and glucose seem to benefit most from the added organic content, showing strong signal increases. Malic acid on the other hand, is detected at slightly higher signal intensities when spraying the DAs in pure aqueous solutions (Fig. 1C and D). Additional solvent ratios (70:30 and 90:10 ACN: H_2_O) were evaluated under exploratory conditions (1 mg/mL 4-APEBA instead of 0.5 mg/mL) and showed no improvement of signal intensities over 50:50 ACN: H_2_O (Supplementary Figure S2). The chosen mixture of 50:50 ACN: H_2_O provided the best compromise for the intended analyses, yielding consistent results across various metabolites and metabolite classes while maintaining tissue integrity. For optimization, we set a focus on small polar carboxylic acids, such as TCA cycle intermediates, and selected low-weight molecular compounds with potential biological implications like pyruvaldehyde. Although we demonstrate only a few selected metabolites, we want to highlight that varying metabolites may require selected optimizations. For example, malic acid, which benefited from higher water content, compared to aspartic acid, which benefited from higher concentrations of 4-APEBA. These metabolites were influenced differently by parameter modifications as discussed above, albeit with no pronounced opposing effects between conditions, but it should be taken into consideration when applying the method for targeted analysis. The finalized workflow is depicted in Supplementary Figure S3.

**Fig. 2:**
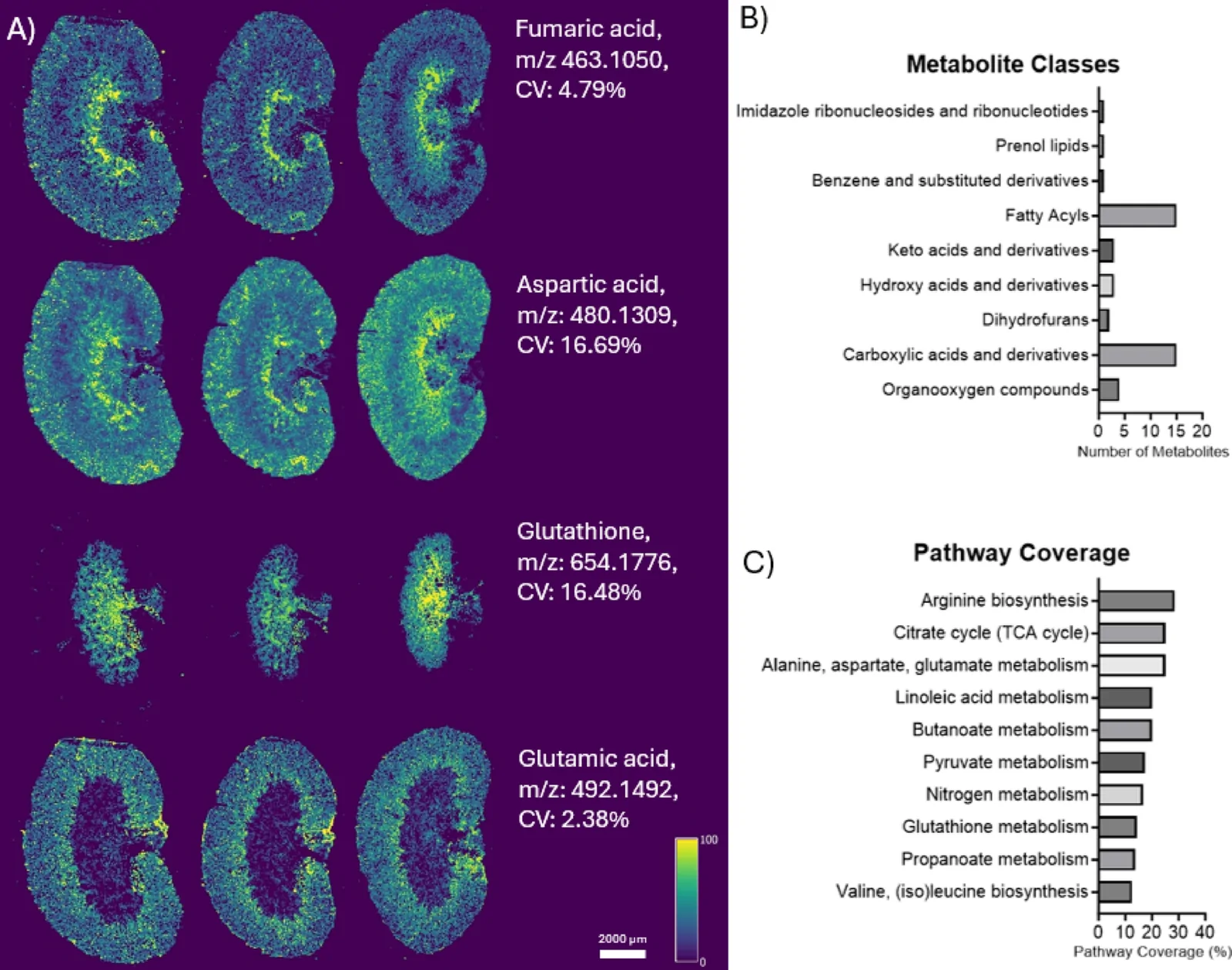
Method performance assessment of OTCD-MALDI-MSI. **A**) Reproducibility assessment across three biological replicates of kidney sections. Comparative ion images of selected metabolites and their coefficients of variation (CVs) derived from ROI-based signal extraction are shown. Signal intensities are indicated by the color scale. **B**) Classification of detected metabolites grouped by metabolite class. **C**) Pathway coverage (%) for the top 10 metabolic pathways represented in the dataset following OTCD-MALDI-MSI analysis. Metabolite annotations were performed using a ± 5 ppm mass error tolerance

Reproducibility has been investigated by measuring triplicate kidney sections prepared with the optimized parameters. Visual comparison of the MALDI images showed highly similar distribution patterns of target metabolites across all replicates (Fig. 2A). Post-MALDI-MSI staining confirmed no major tissue morphology changes across sections (Supplementary Figure S4A) To further evaluate, ROI analysis of the whole kidney section has been performed, and average spectra of the ROIs were generated using LipostarMSI. Resulting average signal intensities were comparable between replicates, with selected metabolites exhibiting coefficients of variation (CVs) below 35%, indicating high reproducibility of the optimized protocol (Fig. 2A).

To assess metabolic coverage achieved with the OTCD protocol, a combined workflow of manual curation and automated data processing using Python was employed. Preferred *m/z*-lists generated by the LipostarMSI peak picking algorithm were extracted, and possible isotopic peak pairs with a 1.998 Da difference were filtered out. The resulting candidate pairs were manually curated to ensure both isotopes are detected in all replicates and exhibit similar signal intensities and spatial distributions. After manual evaluation, peaks were tentatively annotated by calculating the monoisotopic mass and matching against entries from the Human Metabolome Database (HMDB). The data was further filtered based on the presence of functional groups (carboxylic acids, carbonyls) and origin listed in HMDB. This combined workflow led to 46 annotations (92 signal peaks) of derivatized metabolites in kidney replicates (Supplementary Table 4). For annotated metabolites, the theoretical *m/z* values of doubly derivatized reaction products were calculated when chemically plausible. No consistent signals matching these calculated masses were observed in the data. Metabolites from 9 compound classes and 16 subclasses were detected, with carboxylic acids and derivatives and fatty acyls being the most represented classes (Fig. 2B). Among the detected classes, metabolites from 32 different pathways are included, with arginine biosynthesis, TCA-cycle, and alanine, aspartate, and glutamate metabolism exhibiting the most complete pathway coverage (Fig. 2C). The observed spatial patterns in the kidney are consistent with previously published results showing differences in energy metabolism in the kidney cortex and medulla. Notably, the characteristic central localization of glutathione, which is a well-known antioxidant that has a crucial role in cellular protection, is a recurrent pattern we have highlighted in a previous study [10, 11]. In addition, the spatial distributions of other metabolites in our dataset are in agreement with established renal physiology. For example, glutamic acid was primarily detected in the cortical region. In proximal tubular cells, which are primarily located in the cortex, glutamine is taken up and deamidated into glutamate, which is potentially reflected in our data [40].

Metabolic coverage achieved using OTCD was also compared to traditional MALDI matrix workflows (DHB for positive mode and NEDC for negative mode) on the same sample, with half the tissue derivatized and half the tissue sprayed with the respective matrix. Post-MALDI H&E stains can be found in Supplementary Fig. 4B. For assessment, [M + H] + and [M-H]- masses of the tentatively annotated metabolites in OTCD-treated kidney sections were calculated. Direct comparison between DHB-only and OTCD-treated kidney data demonstrated that DHB provides very limited access to the targeted metabolites, with only 2 detected of the 46 metabolites detected with the OTCD workflow (Supplementary Figure S5A). In contrast, OTCD expanded the detected metabolites, confirming that derivatization improves positive-mode coverage of carbonyl- and carboxyl-containing metabolites. Importantly, metabolites, which are traditionally well detected in traditional workflows using DHB, such as carnitines, which are involved in mitochondrial fatty acid transport and energy metabolism, remain detectable in OTCD datasets (Supplementary Figure S5B). This represents an important advantage of our OTCD workflow, as it extends metabolite coverage in one ionization polarity and thereby provides complementary metabolic information.

Comparison between OTCD results and the previously established NEDC workflows resulted in 17 annotations as [M-H]- (except Hexose: [M + Cl]-) from the OTCD metabolite list. Selected metabolites, such as lactic acid and fumaric acid, show considerable off-tissue signal, potentially reflecting background signals from preparation contaminants. OTCD improved spatial specificity of these signals, with significantly less background interference. To assess the contribution of background signal at the respective *m/z* values, S/N ratios were extracted from single pixels on- and off-tissue. Calculated S/N ratios for off- and on-tissue data are exhibited in Supplementary Table S5, showing S/N benefits when the OTCD method is applied. Additionally, a ratio of average off- and on-tissue signal was calculated to show reduced background contribution when using OTCD (average intensity off-tissue/ average intensity on-tissue*100). Shared metabolites (13 out of 17) show better signal intensity or image quality when measured using NEDC (Supplementary Figure S5 C&D). This data suggests that while NEDC remains advantageous for several abundant metabolites in negative ion mode, OTCD provides complementary molecular coverage and enables positive mode analysis of shared metabolites. In total, several annotations (27 of 46), such as hydroxybutyric acid, heme or α-ketoglutaric acid, were uniquely observed after OTCD and not detected when using only DHB or NEDC, which highlights its complementary value (Supplementary Table 4).

### Dried droplet standard confirmation

Since preliminary in-solution chemical derivatization reaction experiments did not yield consistent results for carboxylic acids, we next confirmed the derivatization of selected metabolite standards in a dried-droplet experiment. Metabolite standards of three different concentrations were spotted onto an ITO-slide, dried and subsequently sprayed with DAs and matrix under the same conditions as applied to tissue. This approach enabled confirmation of reaction products and annotations in a matrix-free environment while minimizing the risk of hydrolysis of intermediates.

**Fig. 3:**
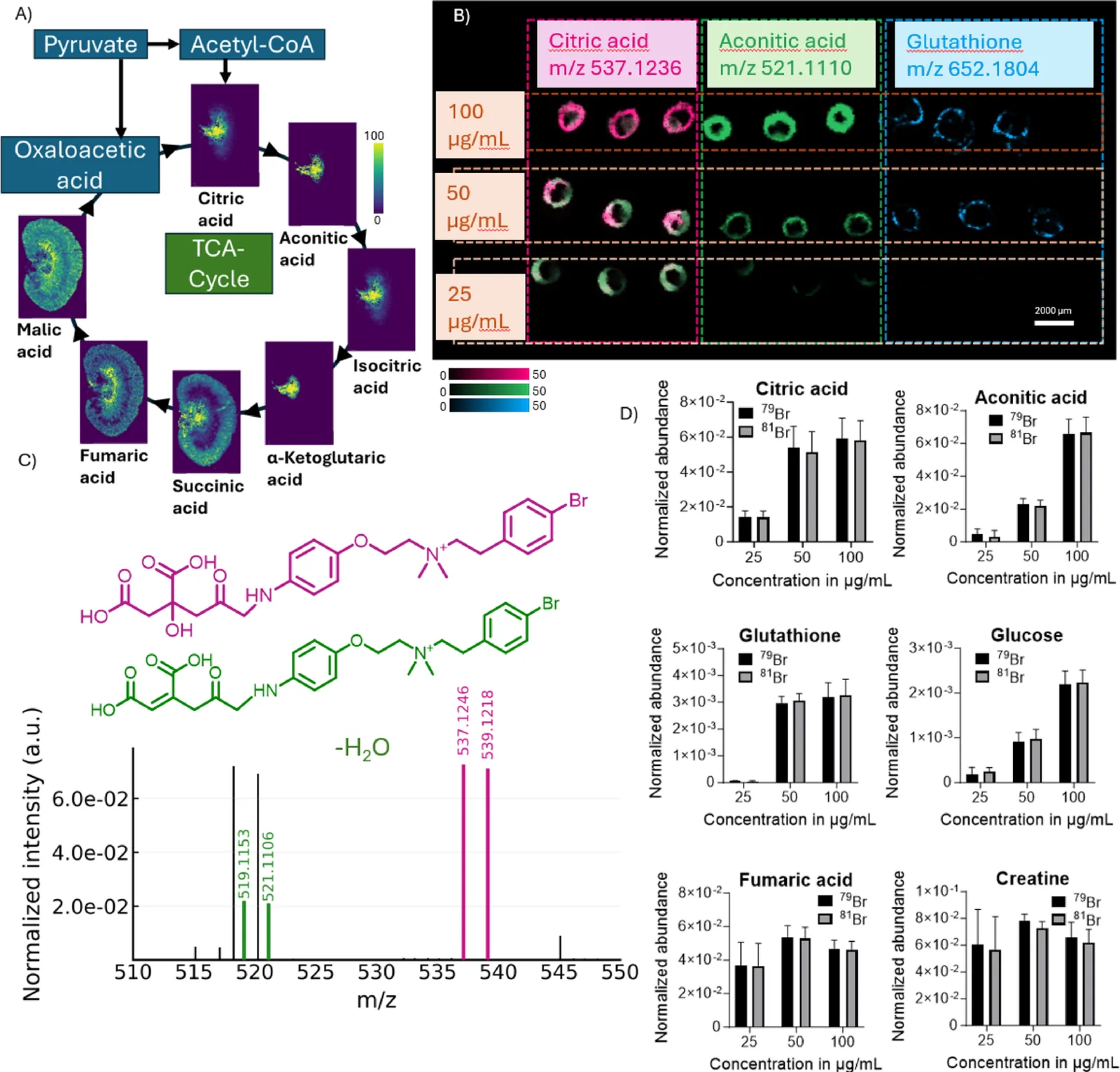
Metabolite annotation and confirmation. **A**) Murine kidney tissue ion images of annotated TCA-cycle metabolites demonstrating coverage of central carbon metabolism. Citric and isocitric acid could not be distinguished due to their structural similarity. Signal intensities are shown according to the color scale. **B**) Ion images of spotted metabolite standards—citric acid (magenta), aconitic acid (green), and glutathione (blue)—applied in triplicate at three different concentrations (100, 50, and 25 µg/mL). Citric acid spots display a mixed signal originating from in-source-fragmentation. Signal intensities are indicated by the color scale. **C**) TIC-normalized mean ROI spectrum of citric acid (100 µg/mL) spots highlighting characteristic bromine isotope peak pairs for derivatized citric acid (m/z 537.1246/539.1218) and aconitic acid (m/z 519.1153/521.1106), the latter attributed to in-source fragmentation. **D**) Quantification of TIC-normalized signal intensities in spotted regions (mean ± standard deviation), including both isotopic reaction products. Metabolite annotations were performed using a ± 5 ppm mass accuracy threshold

Expected derivatization products were detected for all spotted standards, confirming the theoretically calculated mass incurred from the derivatization reaction and providing proof of principle (Supplementary Figure S6). Most metabolites from the TCA cycle were confirmed using this approach (Fig. 3A). Proposed reactions are displayed in Supplementary Figure S7. During dried-droplets experiments, several standards showed minor evidence of in-source fragmentation, resulting in peaks that overlap with the expected masses of other spotted standards. This effect was observed to be minimal for most detected metabolites. Only citric acid produced a strong fragment at *m/z* 519.1153 and 521.1106 corresponding to the expected derivatized masses of aconitic acid, possibly through dehydration in the gas phase similar to the biological formation of aconitic acid from citric acid (Fig. 3B and C) [41]. Overlaps like this complicate metabolite annotations in complex tissue samples, where multiple metabolites are present and the detected signal might originate from more than one analyte, consisting of fragments, intact metabolites, or a combination of both. This limitation is inherent to MALDI-MSI and cannot be fully resolved within the present study [42].

Standards were spotted in three different concentrations, and signal intensities were generally increased at higher concentrations; however, the response was not strictly linear for all measured standards, as observed for fumaric acid and glutathione in Fig. 3D. This is commonly seen in MALDI experiments and could be attributed to inhomogeneous spotting, inhomogeneous crystallization, especially in standard experiments, and ion suppression [43]. Non-derivatized forms of the spotted metabolites were not detected in this experiment, hinting at a high derivatization yield. However, it must be noted that non-derivatized pyruvate and lactic acid were outside of the mass range analyzed and so could not be verified.

Stability of derivatization products was investigated by analyzing spotted analytical standards on three respective days (0 h, 24 h and 48-hour timepoints) and comparing the average signal-to-noise (S/N)-ratio. Due to heterogeneous spot morphology, three areas of each spot were analyzed and averaged. Out of 12 spotted metabolic standards, 8 show a coefficient of variation (CV) below 35%, indicating acceptable short-term stability over the investigated time period (Supplementary Table 1). The remaining four compounds (aconitic acid, creatine, succinic acid and pantothenic acid) showed higher variability between the days, which may be attributed to differences in crystallization and spot morphology, as they showed a strong coffee-ring effect upon drying. Overall, these results support sufficient short-term stability of the derivatization products, in line with previous research [35]. During this evaluation, signals consistent with 1-ethyl-3-(3-dimethylaminopropyl)urea (EDU), a known EDC-related hydrolysis byproduct/intermediate, were observed [44] (Supplementary Figure S8 A&B). Similar signals were observed on tissue. Notably, this signal is also detected outside of the tissue or spotted standard area, indicating DHB has a significant effect on the formation of this byproduct, as it also reacts with EDC/4-APEBA and is applied in aqueous solutions. Similar signals can also be found in previously published EDC/4-APEBA datasets, indicating the formation of this byproduct is inherent to the reaction chemistry rather than specific to this workflow. EDU did not interfere with the annotated compounds.

The standard data were screened to evaluate the occurrence of doubly derivatized products. Corresponding product signals were only detected in the citric acid standard at less than 0.5% of the S/N-ratio of the corresponding singly derivatized ion. No doubly derivatized species were found in other standards, indicating that multiple derivatization events contribute only negligibly under the applied conditions. Additionally, MS/MS was recorded for all standards (*n* = 12) at the highest concentration spotted, as well as directly on tissue after MALDI-MSI. The obtained spectra were dominated by recurring fragment ions that have been described in literature previously [45], such as of *m/z* 58.07 [C_3_H_8_N]^+^, *m/z* 72.08 [C_4_H_10_N]^+^, *m/z* 104.06 [C_5_H_14_NO]^+^ and *m/z* 182.98 [C_8_H_8_Br]^+^, which were observed across different derivatized analytes in standard- and on-tissue data, indicating that fragmentation is primarily driven by the derivatization moiety rather than the specific metabolite (Supplementary Figure S8 C&D). Therefore, the on-tissue MS/MS spectra provide additional evidence for the detection of 4-APEBA-derived products, but, in the absence of analyte-specific fragments, they do not confirm metabolite identities.

To estimate the surface reaction yield, deuterated creatine (d_3_-creatine) was used as a standard detectable both in its native form with only DHB and in its derivatized form. In control measurements without derivatization, the [M + H] + ion of d_3_-creatine (*m/z* 135.0956) was clearly detected. In measurements with derivatization, mainly the derivatized ions (*m/z* 479.1849 and *m/z* 481.1829) were detected. The estimated reaction yield was calculated by comparing the intensity of the [M + H] + ion in matrix controls and derivatized samples. The residual signal corresponded to 2.07% of the control signal, indicating an estimated surface reaction yield of 98%. A comparable estimation for carboxylic acids could not be performed, as no metabolites were confidently detected in both protonated ionization with DHB-only experiments and their derivatized form upon OTCD preparation. This highlights the ability of OTCD to broaden access to metabolites in a single polarity, enabling simultaneous detection of carboxylic acids alongside metabolites that are inherently detectable in positive ion mode.

### Workflow application to metastatic tissue

To demonstrate that the optimized on-tissue derivatization protocol is broadly applicable across tissue types and biologically relevant settings, we applied our approach to two metastatic tissues that differed in tumor size and morphology. For each model, one biological specimen was analyzed using serial sections as technical replicates. Accordingly, these experiments were intended as a proof-of-applicability of the workflow and are interpreted in the context of previously reported metabolic alterations in tumors.

**Fig. 4:**
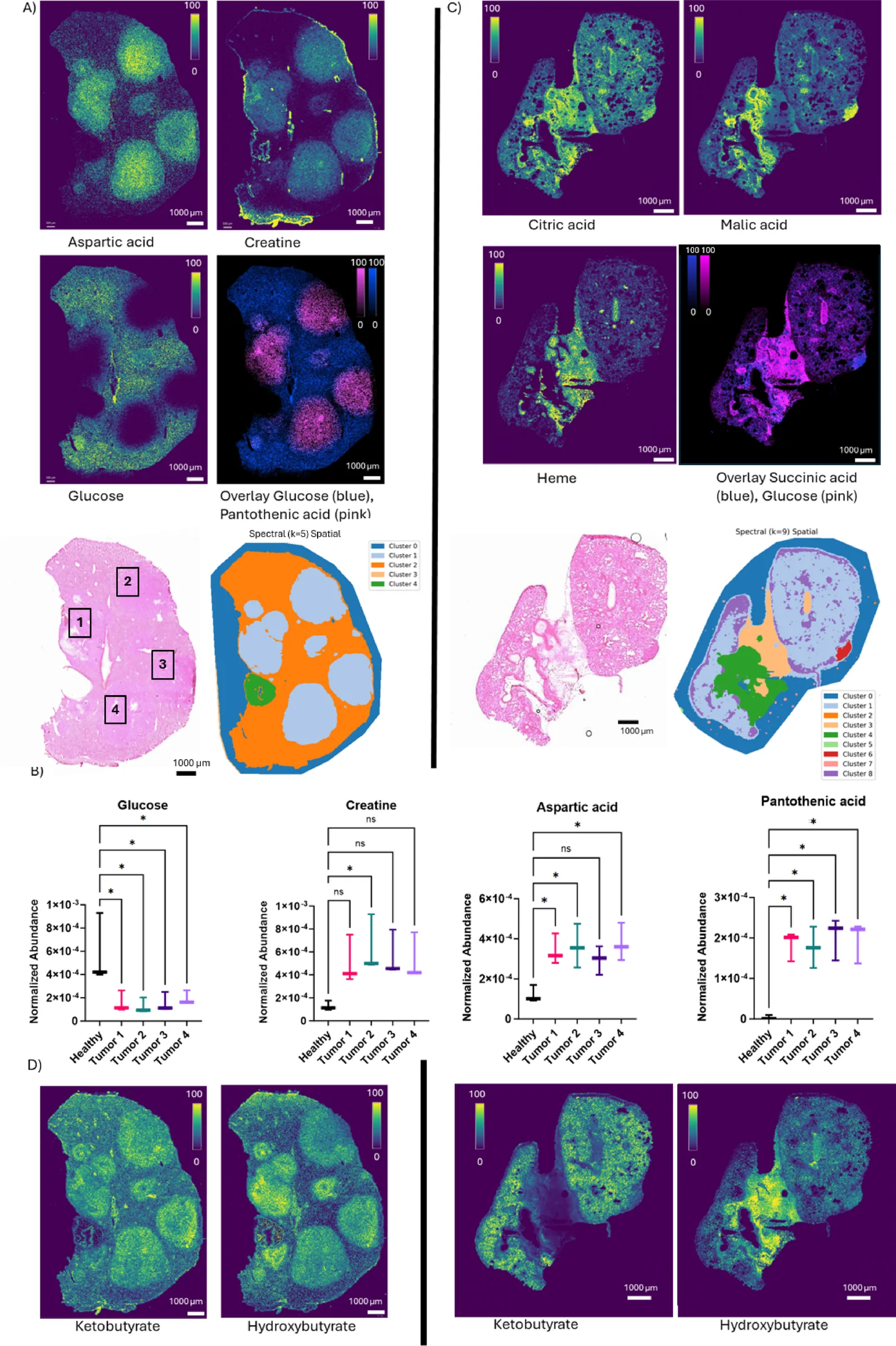
OTCD enables spatial metabolic profiling of metastatic tissues. **A**) Exemplary ion images of derivatized metabolites in a metastatic liver tissue section, highlighting heterogeneous spatial distributions across tumor and adjacent healthy regions. Corresponding H&E staining and spectral clustering results overlayed on a spatial image illustrate anatomical context and data-driven tissue classification. Signal intensities are shown according to the color scale. **B**) ROI-based analysis of tumor versus healthy regions in the liver model from three replicate sections, revealing metabolic differences, visualized as boxplots. Tumor areas are annotated on the H&E reference image. Comparisons were performed between four tumor ROIs and healthy surrounding tissue within each of three serial sections derived from a single animal (*n* = 1), representing technical/pseudo-replicates, and providing a descriptive p-value. Metabolite annotations were assigned using a ± 5 ppm mass tolerance. **C**) Exemplary ion images of metastatic lung tissue depicting differential metabolite abundance in tumor foci relative to surrounding parenchyma, with associated H&E and spectral segmentation images. **D**) Ion images of ketobutyrate and hydroxybutyrate in metastatic lung and liver tissue. Signal intensities are indicated by the color scale

The first evaluated sample was a patient-derived xenograft (PDX) melanoma liver metastasis with extensive and clearly delineated tumor regions, offering a well-controlled model to assess the method in metastatic tissue. The derivatization protocol produced strong signals from numerous metabolites, enabling the tentative annotation of over 70 compounds consistently across replicates (Supplementary Table 6). A subset of metabolites exhibited heterogeneous spatial patterns across the tumor and surrounding liver tissue. As shown in Fig. 4A, certain metabolites showed higher relative signal intensity in tumor regions, whereas others were more abundant in healthy neighboring areas. Still others displayed widespread distribution with little regional specificity (Supplementary Figure S9). ROI-based analysis revealed distinct alterations of the detected molecular profiles, including creatine, aspartic acid, and pantothenic acid, which show a higher relative signal intensity in tumor tissue, while hexose intensities were reduced relative to surrounding healthy regions (Fig. 4B). Collectively, these observations underscore the strength of OTCD in uncovering spatial metabolic heterogeneity within tumor microenvironments.

The spatial variation in metabolite abundance was compared to the literature to evaluate the biological plausibility of observed spatial patterns using OTCD and is in line with the recognized metabolic heterogeneity of tumors, therefore potentially reflecting biological changes. Several observations consistent with hallmarks of glycolytic reprogramming were made [46, 47], including lower hexose and elevated lactic acid signal intensities in tumor regions (Supplementary Figure S9). Although the latter shows minor matrix contribution, the shift of normalized abundances of selected metabolites may support increased glycolytic flux in metastatic tissue. Additionally, lower abundances of TCA-cycle intermediates such as fumaric and malic acid, in comparison to the surrounding healthy tissue, could point toward altered mitochondrial metabolism and reduced oxidative phosphorylation [48]. Small organic acids from the TCA cycle can often suffer from matrix and background interference when analyzed without OTCD. Using our optimized workflow, we were able to visualize distributions of metabolites like fumaric acid on tissue, which we typically observe in the matrix background at relevantly similar signal intensities in imaging experiments, leading to possible ambiguity of metabolite presence against background noise (examples shown in Supplementary Figure S5). Pantothenic acid shows high relative signal intensity in the tumor areas in our datasets, similar to reports in breast tumor localizations (Fig. 4B) [49].

For the second model, lung tissue from a melanoma cell line xenograft mouse was examined, revealing a small yet well-defined metastatic focus embedded within normal parenchyma. Although the lung presents substantial anatomical and metabolic complexity, and the confirmed tumor area was limited, OTCD yielded robust spatially resolved metabolite images and facilitated the tentative annotation of more than 75 compounds (Supplementary Figure S10, Supplementary Table 7). Similar to the liver model, tumor regions exhibited increased relative signal intensities of TCA-cycle metabolites such as citric and malic acid, while hexose displayed reduced abundance, compared to the surrounding healthy lung tissues (Fig. 4C). Heterogeneous metabolite distributions were also detected in the surrounding lung parenchyma, supported by the clear detection of the heme group (Fig. 4C). In addition to carboxylate metabolites, the workflow also enabled the simultaneous detection of several aldehydes such as 4-hydroxynonenal (Supplementary Figure S10).

In both models, OTCD enabled the detection of previously not reliably detected metabolites using NEDC or DHB. In particular, we have highlighted ketobutyrate, which was previously only detected at very low abundance using DHB, and hydroxybutyrate, which was previously undetected, were consistently measured using the OTCD workflow (Fig. 4D). In our liver datasets, ketobutyrate shows higher signal intensities within tumor lesions, whereas in the lung, higher signal intensities are shown in the surrounding parenchyma. Hydroxybutyrate, a byproduct of endogenous cysteine synthesis that was previously undetected, has been suggested as a potential early biomarker for insulin resistance [50] and is involved in the regulation of muscular fatigue [51]. In our datasets, differential abundance of hydroxybutyrate between tumor and non-tumor tissue was only observed in the liver data; however, consistent detection was achieved in kidney, liver and lung. Across both case studies, unsupervised segmentation showed good spatial agreement with the pathologist-annotated H&E tumor regions, with DICE coefficients of 0.84 for the liver and 0.82 for the lung model. It therefore reliably distinguished tumor from non-tumor regions, indicating robust metabolic contrast independent of the analysis method. Spatial segmentation maps and UMAP visualizations, along with validation metrics, are provided in Fig. 4A and C, and Supplementary Figure S11 & S12. Optimal k-values are calculated; however, due to the complexity of the metastatic tissue models of the liver and lung, higher k-values are exhibited in this study to display the impact the methodology can provide with less abundant featured areas. Lower intensity metabolites or smaller feature area segments may be lost if higher k-values are not explored. Quantitative agreement with histopathological annotated post-MALDI H&E images was assessed using confusion matrices, spatial overlap maps and DICE co-efficient (Supplementary Figure S13).

## Discussion

Metabolism plays a critical role in how tumor cells proliferate and adapt to become opportunistic for increasing metastatic potential, and by using spatial metabolomics, tumor environments and microenvironments can be explored for differential localizations of metabolites of interest. Applying our OTCD workflow to detect metabolites adds beneficial value for exploratory metabolomics due to the isotopic confirmations possible and increased coverage in one polarity. Preliminary testing showed that the reaction may not be suitable for in-solution analyses, as only one moiety (carbonyls for aldehydes/ ketones) was successfully reacting, while carboxylic acids failed to produce reaction products. EDC activation for carboxylic acid derivatization can be deduced as a limiting factor due to its instability. The compound-specific reaction optima of EDC are reported to be very pH-dependent. Intermediates formed between carboxylic acids and EDC in the activation step are prone to hydrolysis at non-optimal pH conditions, and may be the cause of an unsuccessful two-step reaction in-solution [52]. However, as fine pH adjustments are not directly translatable to on-tissue derivatization due to background effects and the complex microenvironment of the tissue, no further optimization in solution was performed and this mechanism remains hypothetical. Instead, we focused on establishing the spraying protocol directly on tissue and dried standard spots.

For this, optimization of the application of 4-APEBA and EDC using a non-heated nozzle sprayer was carried out, and better results were achieved by lowering DA concentrations compared to other methods. Lowering DA concentration reduced base peak intensity and ion accumulation in the Orbitrap and thereby minimized ion-suppression and analyzer oversaturation without sacrificing metabolite coverage or sensitivity. Individual metabolites responded differently to parameter changes (e.g. malic acid and aspartic acid benefited from higher DA loads, whereas others, such as pyruvaldehyde, remained unaffected), underscoring the analyte-specific nature of OTCD optimization. The chosen parameters yield a balanced compromise for robust detection of the intended polar metabolite targets on mouse tissue.

Similarly, solvent composition had analyte-specific effects on metabolite detection. Overall, a solvent mixture of 50:50 ACN: H_2_O showed favorable results in terms of analytical sensitivity and coverage compared to pure aqueous spraying as suggested in the original protocol. While pure aqueous spraying improved detection of certain analytes like malic acid, likely due to their high water-solubility (558 g/L for malic acid [53]), it caused wet spraying, analyte delocalization, inhomogeneous tissue coverage, and low relative signal intensities of derivatized carboxylic acids. Delocalization and wet spraying results can likely be attributed to the use of a different spraying apparatus. Unlike the one used in literature, it does not allow us to heat the sprayer nozzle for the applied solution and improve the vaporization of the solvents. Here, the addition of ACN supports the vaporization process, leading to a finer, homogeneous spray.

Beyond spraying behavior, solvent composition may also influence the underlying derivatization chemistry. In LC-MS-based derivatization workflows, high water content has been reported to promote hydrolysis of EDC, thereby effectively lowering derivatization yields of carboxylic acids. Partial substitution of water with acetonitrile has been shown to minimize this trend [39]. Based on these insights and widespread use of ACN in metabolomics experiments, we systematically tested the addition of this solvent to the on-tissue derivatization and found that mixed aqueous–organic conditions offer an effective compromise between reaction performance and metabolite coverage.

The combined manual and automated annotation workflow demonstrated that OTCD enables detection of 46 metabolites across multiple compound classes and 32 pathways in kidney tissue. While annotation platforms such as METASPACE provide a powerful and valuable tool for untargeted metabolite annotations, OTCD-derived datasets with characteristic isotopic patterns benefit from chemistry-informed filtering strategies, as used in this study. However, it should be noted that annotations are based on accurate masses from theoretical values and filtered for chemical plausibility. This approach cannot distinguish isomeric or isobaric compounds; therefore, all annotations are putative.

Among the detected metabolic pathways arginine biosynthesis, TCA cycle, and alanine, aspartate, and glutamate metabolism exhibit the most complete pathway coverage. This shows that using our OTCD approach, we are able to detect polar metabolites, which are typically measured in negative ion mode [17], and fatty acids, which are often measured in positive ion mode [54], in one single polarity by attaching a permanent positive charge. This can be a key advantage of OTCD to maximize targeted analysis output from smaller sample inputs. TCA-cycle metabolites are of particular interest in metabolomics analyses, as they have a central position in cellular energy metabolism. Although frequently reported using traditional MALDI matrices, their low molecular weight and interference with low-mass background signals make reliable annotation challenging. By generating a predictable mass shift and a bromine isotopic doublet, our OTCD workflow reduces the likelihood of misidentifications. Alongside the advantages of higher confidence for identifications, the increase in metabolite coverage from one MSI analysis can be beneficial for cancer applications, as some preclinical and clinical models suffer from low sample availability and can limit the output of information gained.

Our results demonstrate that applying an optimized EDC/4-APEBA protocol to murine tissue for spatial metabolomics, increase in metabolite coverage and image quality with doublet-based data analysis enhancing annotation confidence can be achieved.

Application of the workflow to two metastatic tumor tissues underscores the strength of OTCD in uncovering spatial metabolic heterogeneity within tumor microenvironments. Utilizing the OTCD method, multiple cancer-relevant metabolites were detected in liver metastatic tumors, which coincide with current literature. Markedly, small acidic metabolites like fumaric acid and malic acid were detected on tissue with minimal background interference. This is beneficial in spatial analysis of cancer tissues, as they are highly regulated intermediates of tumor development [55]. Detection of other high intensity metabolites such as pantothenic acid may also hint at the accumulation of energy-related metabolites for alternative energy pathways. Pantothenic acid is a precursor of coenzyme A and has been reported to accumulate in c-MYC high areas of breast cancer tumors, where it supports tumor growth and energy metabolism [49]. This is also seen in other cancer models and is indicated as a mechanism to circumvent protective measurements for continued tumor growth. Given the limited number of biological replicates for this methodology study, these interpretations remain suggestive until further correlating analysis or interruptive studies can be performed.

In the lung metastasis model, observed metabolic alterations mirror the metabolic reprogramming noted in the liver metastasis model, indicating shifts in central carbon metabolism and enhanced energy production to support tumor growth [48]. Elevated glutathione signal intensities in the metastatic region further align with emerging evidence highlighting mitochondrial glutathione as a driver of lung metastatic colonization [56]. Heterogeneous metabolite distributions in the surrounding lung parenchyma further demonstrate the suitability of OTCD for probing metabolic changes in diverse tissues with structurally complex and blood-rich backgrounds. Due to this highly suppressive background, these can be very challenging tissues, including cardiac tissue, for successful small-intensity metabolite imaging. In addition to carboxylate metabolites, aldehydes such as 4-hydroxynonenal were successfully derivatized. This reactive aldehyde has been reported to be elevated in patients with chronic lung diseases like chronic obstructive pulmonary disease (COPD) [57] and was previously visualized in diabetic kidney tissue using GT [58]. Unlike this single-class reagent, our workflow simultaneously derivatizes aldehydes and carboxylic acids and thereby expands the range of detectable metabolite species in a single measurement. As lung tissue is infrequently analyzed by MALDI-MSI due to its fragile architecture and pronounced heterogeneity, these results highlight the broad applicability of OTCD to underrepresented and complex specimen types. Furthermore, the optimized OTCD workflow expands access to biologically relevant molecules, that are difficult to detect using conventional MALDI-MSI workflows. The reliable detection of metabolites with clinical associations like hydroxybutyrate and ketobutyrate demonstrates the capabilities of OTCD to enable spatial analysis of biologically relevant metabolites, that may otherwise remain inaccessible or challenging to detect in standard workflows. Ketobutyrate, which is linked to amino acid catabolism, has been previously associated with metabolic reprogramming in renal cell carcinoma (RCC) and proposed as a potential biomarker for the aggressive subtype clear cell RCC [59], demonstrating the clinical relevance of investigating this compound. Given the cross-cancer and cross-species nature of this comparison, this link should be regarded as a suggestive association that ketobutyrate can be a metabolic biomarker of interest rather than evidence. A shared biological role would require further investigation in human tissue of matched tumor models.

While the observed spatial trends in both models align with known metabolic changes in metastases, it should be noted that changes in relative signal intensity do not necessarily reflect changes in metabolite concentrations between tissue regions but can also arise from matrix effects and differences in local ion suppression. Integrating OTCD with stable isotope tracing strategies or quantitative MSI in future work could enable direct measurement of metabolic fluxes within intact metastases, providing deeper insight into metabolic adaptations in complex tissue microenvironments [11, 60–62]. In addition, both applications models used are based on melanoma disease, but one is generated from an immortalized cell model and one from a PDX cell model. As these models are distinctly different, the discussions generated are solely based on the detection of spatial metabolites and their signal intensities. Further biological investigations would be beneficial for deeper insights into potential mechanistic or targeted biochemical pathway analysis.

Collectively, these results demonstrate that OTCD spatial metabolomics supports biologically meaningful insights in well-defined metastatic tissues as well as in more challenging samples with limited tumor involvement. While previous studies have been focused on agar, plant or selected human samples, this work expands the insights possible and technical applicability of the OTCD workflow to preclinical cancer mouse models and complex tumor and tissue microenvironments. Spatial patterns were highly reproducible across replicates and corresponded closely with H&E histopathology, confirming no major spatial delocalization of metabolites is observed at the pixel size investigated (20–25 μm). While metabolite assignments remain tentative due to possible interference from in-source fragmentation or isomers, our findings show that OTCD effectively captures the ability to detect disease-associated metabolic alterations and can advance our capabilities of understanding metabolic diversity within tumor microenvironments.

## Conclusion

Overall, on-tissue chemical derivatization represents a promising strategy to overcome current constraints in spatial metabolomics, particularly the annotation confidence and coverage of endogenous metabolites in MALDI-MSI for smaller input sample amounts. In this study, we systematically optimized and displayed an OTCD workflow using EDC and 4-APEBA to improve detection of carboxyl- and carbonyl-containing metabolites across multiple murine tissue types. Optimization of experimental parameters enabled reproducible ion signals and high-confidence metabolite annotation facilitated by diagnostic bromine isotope patterns in derivatized products.

When applied to two metastatic tissue models, the approach revealed spatially distinct metabolic features between tumor and surrounding tissue microenvironments, including alterations consistent with rewired energy metabolism. Notably, clear metabolic contrast was achieved in both large metastatic regions and smaller, structurally challenging foci, demonstrating the broad applicability of the method.

Remaining limitations in unambiguous annotation, arising from potential in-source fragmentation and spectral overlap, may be addressed through future advances in derivatization strategies, instrument performance, and analytical software. Nevertheless, the present results establish a strong basis for robust spatial metabolomic characterization of complex tissue samples.

In conclusion, this work demonstrates the applicability and reliability of OTCD in murine cancer models and highlights its potential to uncover metabolic heterogeneity in health and disease, paving the way for expanded use in spatial metabolomics studies for cancer research.

## Supplementary Information

Supplementary Material 1

Supplementary Material 2

## Data Availability

All data supporting the findings of this study, including the measurement data, can be made publicly available or made available by request to the authors upon publication.

## Abbreviations

4-APEBA: 4-(2-((4-bromophenethyl)dimethylammonio)ethoxy)benzenaminium dibromide
ACN: Acetonitrile
AGC: Automatic gain control
AMPP: 1-(4-(aminomethyl)phenyl)pyridin-1-ium chloride
BMFTR: Bundesministerium für Forschung, Technologie und Raumfaht
CV: Coefficient of variation
DA: Derivatization agent
DHB: 2,5-dihydrobenzoic acid
EDC: 1-ethyl-3-(3-(dimentyhlamino)propyl)carbodiimide
EDU: 1-ethyl-3-(3-dimethylaminopropyl)urea
EtOH: Ethanol
FMP-10: 2-fluoro-1-methylpyridinium
H&E: Hematoxylin and eosin
HMDB: Human Metabolome Database
HPF: High-pressure funnel
IVC: Individually ventilated cage system
LC-MS/MS: Liquid chromatography-tandem mass spectrometry
LPF: Low-pressure funnel
MALDI-MSI: Matrix-assisted laser desorption/ionization mass spectrometry imaging
MeOH: Methanol
MS/MS: Tandem mass spectrometry
NEDC: N-(1-naphthyl)ethylenediamine dihydrochloride
NXG: NOD-Prkdcscid-IL2rgTm1/Rj
OTCD: On-tissue chemical derivatization
PBS: Phosphate buffered saline
PCA: Principal component analysis
PDX: Patient derived xenograft
ROI: Region of interest
S/N: Signal-to-noise
SMM: Small molecule metabolite
TCA: Tricarboxylic acid
TFA: Trifluoroacetic acid
TIC: Total ion current
TierSchG: Tierschutzgesetz
TMPA: N, N,N-trimethyl-2-(piperazin-1-yl)¬ethan-1-aminium iodide
UMAP: Uniform manifold approximation and projection
VersTierMeldV: Versuchstier-Meldeverordnung
ZTL: Zentrales Tierlaboratorium
